# Genetic analysis of wheat ear architecture in F2 hybrid of tetraploid wheats Triticum aethiopicum and T. carthlicum and its computer phenotyping

**DOI:** 10.18699/vjgb-26-55

**Published:** 2026-05

**Authors:** Yu.V. Kruchinina, E.G. Komyshev, M.A. Genaev, V.S. Koval, D.A. Afonnikov, N.P. Goncharov

**Affiliations:** Institute of Cytology and Genetics of the Siberian Branch of the Russian Academy of Sciences, Novosibirsk, Russia; Institute of Cytology and Genetics of the Siberian Branch of the Russian Academy of Sciences, Novosibirsk, Russia Kurchatov Genomic Center of ICG SB RAS, Novosibirsk, Russia; Institute of Cytology and Genetics of the Siberian Branch of the Russian Academy of Sciences, Novosibirsk, Russia Kurchatov Genomic Center of ICG SB RAS, Novosibirsk, Russia; Institute of Cytology and Genetics of the Siberian Branch of the Russian Academy of Sciences, Novosibirsk, Russia Kurchatov Genomic Center of ICG SB RAS, Novosibirsk, Russia; Institute of Cytology and Genetics of the Siberian Branch of the Russian Academy of Sciences, Novosibirsk, Russia Kurchatov Genomic Center of ICG SB RAS, Novosibirsk, Russia Novosibirsk State University, Novosibirsk, Russia; Institute of Cytology and Genetics of the Siberian Branch of the Russian Academy of Sciences, Novosibirsk, Russia

**Keywords:** tetraploid wheat, Triticum aethiopicum, T. carthlicum, genetic analysis, hybridization, cleavage, digital phenotyping, machine learning, тетраплоидные пшеницы, Triticum aethiopicum, T. carthlicum, генетический анализ, гибридизация, расщепление, цифровое фенотипирование, машинное обучение

## Abstract

A comprehensive description of plant phenotypes of certain taxa is an important task when describing genera and species, as well as when setting their natural taxonomies. The development of modern technologies of effective phenotyping makes it possible to obtain a large amount of data with a quantitative and/or qualitative description of various traits in plants, mainly based on the analysis of their digital images. The study compared the results of the F2 hybrids assessment – visually and using machine learning methods – of two endemic tetraploid (2n = 4x = 28) wheat species which are Ethiopian wheat (Triticum aethiopicum Jakubz.) and Kartalian or Dika wheat (T. carthlicum Nevski). In the latter case, it is proposed to use the method of a mixture of Gaussian (normal) distributions in plant morphometry in order to identify groups that differ in character values. Most taxonomically important (species-specific) traits are controlled oligogenically and have a clear phenotypic manifestation, so hybridological analysis was an indispensable and basic type of analysis for subsequent detailed phenotyping of wheat spikes using machine-learning methods. According to a number of criteria, the estimates of patterns of inheritance obtained by different methods coincide. Based on the conducted research, we can state that the trait “tetraaristatum” (the presence of awns on both flower and spike glumes) is species-specific (taxonomically important) for T. carthlicum and it can be effectively used for taxonomic purposes both in carrying out hybridological analysis and in experiments using machine learning. Such a species-specific character is the “character (type) of awnedness” for T. aethiopicum. Our study demonstrates that a combination of automatic phenotyping methods and a model of a mixture of Gaussian distributions can, in principle, lead to an automatic analysis of the allocation of classes in F2 hybrids. It allows, in turn, to detect the presence of genes associated with species-specific traits of wheat plants. Further, the improvement of the applied artificial intelligence (AI) algorithms is required.

## Abstract (ru)

Всестороннее описание фенотипов растений конкретных таксонов является важной задачей при описаниях родов, видов и построениях их естественных классификаций. Развитие современных технологий эффективного фенотипирования позволяет получать большое количество данных с количественным и/или качественным описанием различных признаков у растений, преимущественно на основе анализа их цифровых изображений. В исследовании проведено сравнение результатов оценки расщепления в F2 гибридов двух эндемичных тетраплоидных (2n = 4x = 28) видов пшениц – пшеницы эфиопской (Triticum aethiopicum Jakubz.) и пшеницы карталинской (T. carthlicum Nevski), полученных визуально и с помощью методов машинного обучения. В последнем случае для анализа расщеплений предложено использовать метод смеси гауссовых (нормальных) распределений в морфометрии растений для того, чтобы выделить группы, которые различаются по значениям признаков. Ввиду того что большинство таксономически значимых (видообразующих) признаков у пшениц контролируется олигогенно и имеет четкое фенотипическое проявление, гибридологический анализ был незаменимым и основным видом анализа для последующего детального фенотипирования колосьев видов пшениц с использованием методов машинного обучения. По ряду признаков оценки характера наследования таких признаков, полученные разными методами, совпадают. На основании результатов проведенного исследования мы можем утверждать, что признак «тетраостость» (наличие остей одновременно на цветковой и колосковой чешуях) видоспецифический (таксономически значим) для T. carthlicum и он может быть эффективно использован исследователями в таксономических целях как при проведении гибридологического анализа, так и в экспериментах с использованием машинного обучениям. Для T. aethiopicum таким видоспецифическим признаком является «характер (тип) остистости». Наша работа демонстрирует, что комбинация методов автоматического фенотипирования, модели смеси гауссовых распределений в принципе позволит проводить автоматический анализ выделения классов в F2 гибридов, различающихся по величинам фенотипических признаков. Это позволит, в свою очередь, выявлять наличие генов, ассоциированных с видоспецифическими признаками растений. Тем не менее требуется совершенствование применяемых алгоритмов искусственного интеллекта.

## Introduction

A comprehensive description of the phenotypes of plants in specific taxa is an important task for effectively describing of species and constructing natural taxonomy (Hodač et al., 2023; Ran et al., 2024). However, this is a labor-consuming task. In some cases, there is very little information about a particular species, or the species is very rare (Goncharov, Adonina, 2024). In other cases, there is so much information that researchers disagree on the taxonomy of specific accession (Lyapunova, 2021). In addition, information on the species and volumes of genus varies significantly in a number of taxonomies that are still used today. Examples include Solanum L. by S.M. Bukasov (1971) and J. Hawkes (1963); Aegilops L. by P.M. Zhukovsky (1928) and A. Eig (1929); Triticum L. by N.P. Goncharov (2011) and K. Hammer et al. (2011). Therefore, researchers may experience inaccuracies in the taxonomy of species, even knowing which species-specific (taxonomically important) traits are characteristic of a certain taxon. The solution of this problem is to create a system of species-specific characters.

Using cereals (Poaceae Barnhart) as an experimental material, specifically wheat (genus Triticum), one can encounter all the difficulties described above. In addition, it should be borne in mind that wheat is a polymorphic crop, which implies significant inter- and intraspecific variability (Dorofeev et al., 1979). That is, in the process of studying it, one may encounter the fact that the plants inside the specific species will be phenotypically dissimilar and require conducting an experimental assessment of their species affiliation (Zuev et al., 2019). In such cases, we are talking about the unambiguous taxonomies of lower taxa, such as subspecies and subvarieties (Dorofeev et al., 1979; Goncharov, 2009).

Currently, there are a number of methods that allow to assign a specific wheat accessions to a particular species with a high degree of certainty. Using cytological methods, you can accurately assign an accessions to a specific ploidy group by counting the number of chromosomes (Dolezel et al., 2007), while molecular-biology methods can be used to assign accessions to a specific species (Golovnina et al., 2007). However, the use of molecular methods requires a large amount of information about each species, including a description of speciesspecific genes, their localization, and information about their nucleotide sequence. In wheat, species-specific genes have been described only in all hexaploid species (2n = 6x = 42) (Goncharov, 2011). This is the source of troubles for taxonomists. First, not each species of wheat that has been described so far has a species-specific gene(s). Second, even if a gene(s) is known, there may be difficulties in identifying its nucleotide sequence. Third, in some cases, a gene may be known, as well as its sequence, but its molecular function and phenotypic expression may not be studied. In addition, the information about the inheritance of a particular gene may vary among different authors (URL: http://shigen.nig.ac.jp/wheat/komugi/genes/symbol ClassList.jsp Accepted October 20, 2025). Furthermore, it should be noted that molecular biological methods for analyzing large accessions lead to significant costs.

There are other methods that allow us to clarify information about species-specific genes. One of these methods is hybridological analysis. This method helps us to understand how species-specific traits are inherited in certain wheat species. By analyzing the patterns of inheritance in hybrid populations, we can gain insights into the way traits are passed down.

It should be noted that the development of modern technologies for effective phenotyping allows to obtain a large amount of data with quantitative or qualitative descriptions of various plant characteristics, primarily based on the analysis of digital images (Afonnikov et al., 2016; Awada et al., 2024). The data obtained through the analysis of digital images can be successfully utilized for the taxonomy and classification of plants (Chouhan et al., 2024; Mulugeta et al., 2024).

Approaches based on the analysis of large volumes of data and machine learning are also used to analyze the morphological characteristics of wheat. It has been shown that data obtained using image analysis and machine learning methods can successfully solve problems of the taxonomies of wheat species and their relatives (Martinek, Bednar, 2001; Pronozin et al., 2021; Artemenko et al., 2024; Komyshev et al., 2024). This is a promising area of research on hybrids using methods of bioinformatics, which is currently being developed. So the question remains open of how reliably it is to use characters, the characteristics of which are obtained on the basis of the analysis of digital images, for species taxonomies and how they are inherited.

The purpose of the investigation is to combine the possibilities of the hybridological method with the analysis of the splitting of the hybrid population manually with the analysis of digital characteristics of plants (spikes) to solve the problem of dividing plants into groups carrying different genes of plant traits control. The study is based on plant hybrids obtained by crossing two endemic wheat species, T. aethiopicum Jakubz. and T. carthlicum Nevski

## Materials and methods

Hybridological analysis. The work identified the genotypes of tetraploid (2n = 4x = 28) wheat and their F2 hybrids based on species-specific (taxonomically important) traits (Supplementary Table S1)_1_. Since most of these traits are controlled by oligogenes and have a clear phenotypic manifestation, the hybridological method was an indispensable and primary type of analysis for subsequent detailed phenotyping spikes of wheat species using machine learning methods.

Supplementary Materials are available in the online version of the paper: https://vavilov.elpub.ru/jour/manager/files/Suppl_Kruch_Engl_30_3.pdf


Plant material. The object of study was interspecific hybrids obtained by crossing two endemic tetraploid wheat species ♀T. aethiopicum Jakubz. (k-19301/2) with ♂T. carthlicum Nevski (k-32496). The experiment was produced in spring sowing in the greenhouses of the Breeding and Genetics Complex (BGC) of the Institute of Cytology and Genetics of the Siberian Branch of the Russian Academy of Sciences (Novosibirsk). F1 hybrids were planted and phenotyped in a greenhouse, and F2 hybrids were grown in greenhouses in 2022. 185 F2 hybrids were studied

Phenotyping of spike. Image acquisition of spike was carried out according to the “on a clothespin” protocol de- 1 Supplementary Tables S1–S3 and Figure S1 are available at: https://vavilov.elpub.ru/jour/manager/files/Suppl_Kruch_Engl_30_3.pdf scribed earlier (Genaev et al., 2018, 2019). The spike was fixed in an upright position with a clothespin on a blue background. The shooting was done with a Canon 350D digital camera and an EF-S 18–55 mm f/3.5–5.6 lens. An X- Rite Mini ColorChecker Classic marker (http://xritephoto.com/ colorchecker-targets) was placed in the frame to assess the scale and color calibration. The spikes were shot in four projections. The first projection corresponded to the front (widest) side of the spike, and the remaining projections were obtained by rotating the spike on the clothespin 90°: the second and fourth projections were the side projections, and the third projection was the back projection.

Each spike image was segmented into background, color palette, spike body, and awns using a deep machine learning method developed by us earlier (Artemenko et al., 2024). The obtained masks were used by the WERecognizer program (Genaev et al., 2019) to calculate the morphometric characteristics of the spikes. We considered a reduced set of characters, which included a description of the spike based on a model of two quadrilaterals symmetrized with respect to the axis of the spike, characteristics of the contour of the spike (perimeter, area, roundness, etc.), and the area of the awns in the image (Komyshev et al., 2024). The diagram of the quadrilateral model and the parameters of the spike used are provided in the Supplementary Materials (Fig. S1). In total, 19 characters of the size and shape per spikes were analyzed independently for each projection (Table S2).

Statistical analysis of the signs of colossus. In our work, we assumed that the population of F2 hybrid plants can be divided into classes not only based on qualitative (discrete) characteristics (Table 1), but also in quantitative terms. In the second case, it was assumed that each class of plants is characterized by a distribution of the magnitude of the trait with a different expectation and variance. Thus, the distribution of the character value for the entire accessions is a mixture of two Gaussian distributions with different means and variances (Merezhko, 2005; Rechkin, 2024). It should be noted that the method of Gaussian distribution mixture is used in plant morphometry to identify groups that differ in character values, as well as for taxonomy (Kim et al., 2024; Tiburtini et al., 2025).

**Table 1.**
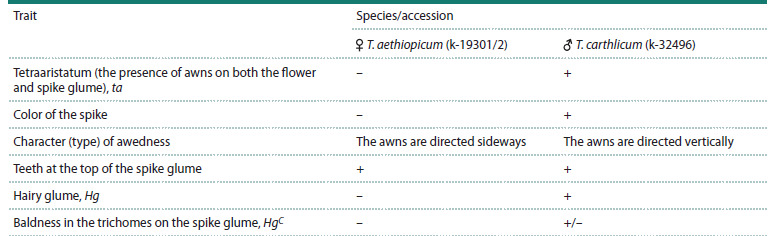
The phenotypic description of the parent forms Note. Trait: – recessive, + dominant.

We used the sklearn.mixture.GaussianMixture function from the scikit-learn v. 1.7 package (https://scikit-learn. org/stable/index.html) to identify mixtures of two Gaussian distributions. This function allows us to analyze a mixture model of Gaussian distributions using the maximum likelihood method, estimating the number and parameters of the distributions in the mixture, and classifying accessions in the dataset based on their belonging to these distributions. In this study, we assumed that the distribution of each character was a mixture of two distributions for each projection of the spike. Using the sklearn.mixture.GaussianMixture function, the mean values, variances, and the number of ears belonging to each distribution were estimated. After determining the number of spikes in each class, a Pearson

χ2 test was performed to check for compliance with the theoretically expected ratio. It was assumed that the observed and AI-generated splits corresponded to the spike traits if the latter was reliable for all four spike projections. The Past v 5.3 package (Hammer Ø. et al., 2001) was used for statistical processing (tests for equality of means and analysis of variance) and data visualization.

## Results

**Fig. 1.**
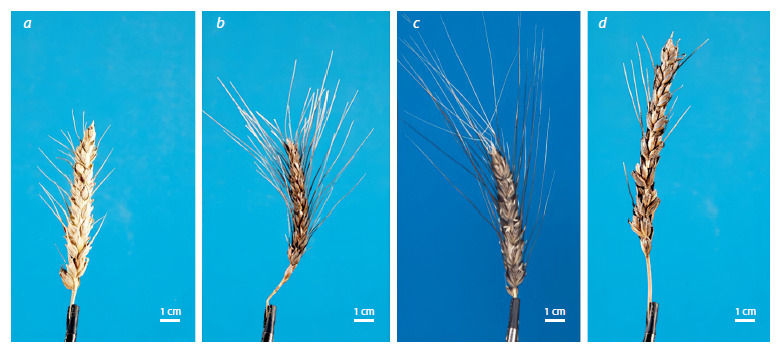
The spikes of the parent species, F1 and F2 hybrids, presented in the same scale and projection. The mother T. aethiopicum (k-19301/2) (a) and the father T. carthlicum (k-32496) (b) plants; the F1 (c) and F2 (d) hybrid plants.

Examples of images of the spikes of the parent species, F1, and F2 hybrids (projection 1) are shown in Figure 1.

**Fig. 2.**
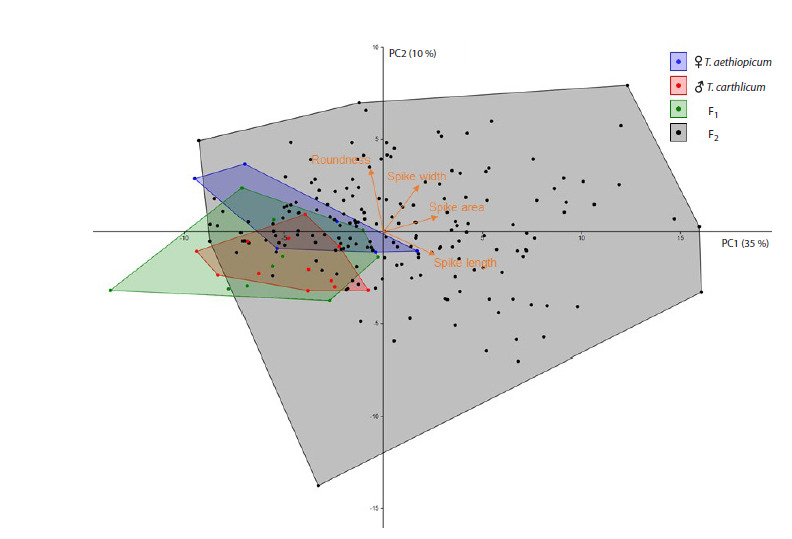
Scatter plot in the space of the first two principal components (PC1, PC2) for the spikes, whose size and shape parameters were independently estimated for the four projections. The symbols are located in the upper right corner. The proportions of the variance accounted for by the first (PC1, horizontal axis) and second (PC2, vertical axis) principal components are given in parentheses. The averaged directions of the projection of the main groups of traits of the spike are indicated by orange arrows.

A principal component analysis (PCA) was performed for four projections to assess the diversity of the “size” and “shape” of the spikes. The results are presented in Figure 2. Of the 76 variables used for the analysis, the first two components accounted for 45 % of the variance, with 35 % accounted for by PC1 and 10 % accounted for by PC2 (Fig. 2). The main contributors to the variability were the traits related to the size of the ear (its length and area). The second principal component of variability is related to the shape of the spike and reflects its roundness. The diagram (Fig. 2) shows that the spikes of the mother, father, and F1 hybrid plants are compactly arranged, occupying partially overlapping areas and demonstrating the similarity of the spike shape/size (Fig. 1a–c). The F2 hybrid plants exhibit significantly greater variability in spike characteristics (primarily in size). It contains a large number of plants with a spike size larger than the average for the first three lines.

In the second stage of our research, we analyzed the F2 hybrid segregation based on the species-specific characteristics of the spikes (Table 1). For each characteristics of traits, we evaluated the reliability of the segregation in the population using proportions that reflect the type of genetic control (3:1, 13:3, 15:1, 61:3, and 63:1) the value of the Pearson χ2 criterion was calculated (Table 2).

**Table 2.**
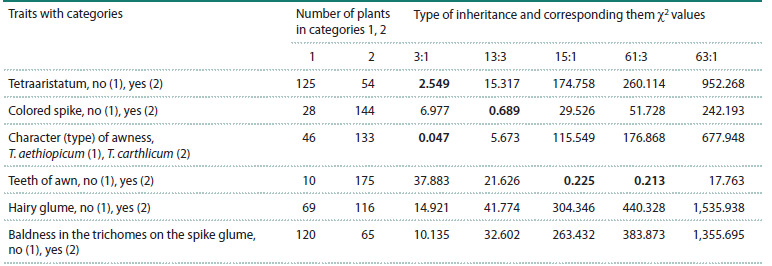
Values of the χ2 statistic for different proportions of occurrence of species-specific spike traits in F2 hybrids of T. aethiopicum and T. carthlicum Note. The left column lists the spike characters and the corresponding category numbers (in parentheses). χ2 0.05 = 3.84. The values of χ2 ratios that do not significantly differ from the corresponding proportions are highlighted in bold.

Based on the results presented in Table 2, it can be concluded that tetraaristatum and the character (type) of awness are split in F2 hybrids in a 3:1 ratio (monogenic inheritance), the presence of spike colored in a 13:3 ratio (digenic inheritance with incomplete dominance), and the teeth of awn in a 15:1 ratio (digenic inheritance). For such traits as the hairy glume and the baldness in the trichomes on the spike glume, no significant similarity was found for the F2 hybrids.

We evaluated 19 spike traits per each F2 hybrid plants that are of interest for identifying those that may produce a split corresponding to specific types of inheritance. We used a mixture model of two Gaussian (normal) distributions to investigate this issue. Each projection was evaluated independently. Since a reliable estimate of the Gaussian distribution parameters depends on the accession size (the number of accessions studied), we decided to limit ourselves to testing the 3:1 ratio in this work. For a total samples of 187 spikes, the theoretically expected number of plant groups is 140 to 47. Table S3 shows the mean values and variances for the first and second group of ears for each character and projection (columns mean1, var1, mean2, var2), the estimated number of spikes in the first and second group (num1, num2), the ratio of the number of spikes (numratio_1vs2), the value of χ2, and the significance level (chisq, chisq_p). It can be seen from the results presented in Table S3 that for two to seven traits in each projection, the ratio of the number of spikes in the two groups is close to 3:1. For example, for the first projection, these traits are the area of the awns (SAA) and the integrity (SSO) of the spike. For the second projection, the width of the spike model and its area (q_ym, q_S), the perimeter of the spike projection contour (SP), its area (SA), the area of the awns (SAA), and the roundness (SRO), etc. It is important to note that only for one character, namely, the “area of the awns” on the image (SAA), a significant split in the ratio of approximately 3:1 is observed for all four projections of the spike. The characteristics of the split for this trait for four projections and estimates of the number of spikes for two groups, χ2, and the probability of the 3:1 monogenic inheritance ratio hypothesis are shown in Table 3.

**Table 3.**
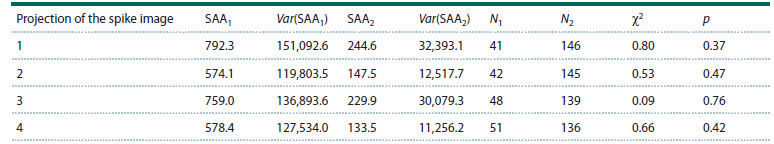
Characteristics of spike splitting in F2 hybrids based on the “area of awns” in the image (SAA) For each spike projection, the estimates of the mean values (SAA1 and SAA2) and variances for the two groups of spikes (Var(SAA1), Var(SAA2)) identified based on the mixture model of two Gaussian distributions are given. Note. N1, N2 are number of accessions of groups of spikes, and p is the probability.

**Fig. 3.**
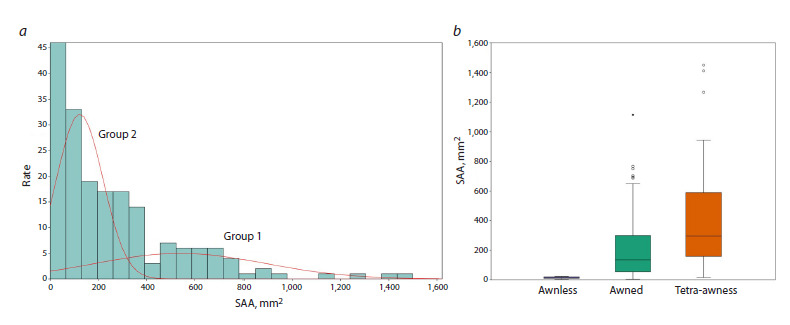
Distribution of the area of the awns. a – histogram of the distribution of the area of the awns on the image (SAA) of the spikes in F2 hybrids (projection No. 4). The X axis shows the value of the trait, and the Y axis shows the number of spikes. The red lines show the probability density distributions for two groups of spikes, namely, those with more (group 2) and fewer (group 1) awns. b – distribution of the area of the awns on the image of the spikes in the 4th projection for the awnless, awned, and tetraaristatum spikes, shown as diagrams. The X axis shows the types of awns, and the Y axis shows the area of the awns.

From the results presented in Table 3, it can be seen that the average values of the spike area are slightly larger for the projection of the front (1) and back (3) sides than for their two lateral projections (2, 4), for both the first and second studied groups. For the group of spikes with a larger area of spikes (and a large number of them, group 1), their number is smaller and varies between 41–51. For a group of plants with a smaller area of awness, the number of spikes varies from 136 to 146. The example of a histogram of the distribution of SAA values for the 4th projection, showing two Gaussian distributions, is shown in Figure 3a

The histogram area is visually divided into two parts: the right part, with fewer plants and a higher SAA value, and the left part, with lower SAA values (more plants) (Fig. 3a). It is in good agreement with the data presented in Table 3. The data presented in Table 3 and in Figure 3a are in agreement with the results of the manual assessment of splitting of spikes in F2 hybrids on the basis of “tetraaristatum” (Table 2). Although, based only on such a parameter as the area of the spikes, it is impossible to determine the tetraaristatum of the spike (the presence of spikes simultaneously on the flower and on spikelet glimes), tetraaristatum spikes generally have a larger number of spines, which leads to an increase in their SAA parameter. This is confirmed by the shift in the area of the awns in tetraploid spikes towards higher values compared to conventional awned spikes (Fig. 3b). We also calculated the average values of the SAA parameter for the four projections of the spikes in the images of the F2 hybrids and performed a test for the equality of the means in three groups of plants: awnless, awned, and tetraaristatum spikes (Table 4).

**Table 4.**
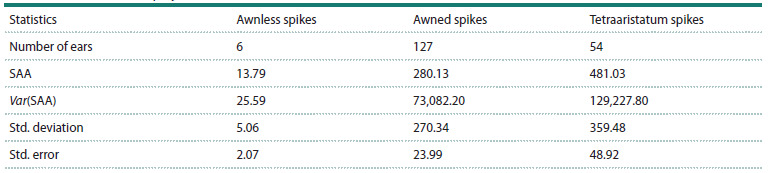
Comparison of the statistics of the distributions of the spikes by the average value of the area of the awns (mm2) in four projections Note. SAA is the area of the spikes in the image; Var is the variance.

Note that the average value of the spinous area is the smallest in spineless plants, the maximum – in tetraaristatum plants, and the intermediate value – in spinous plants, in accordance with the distribution results (Table 4), shown in Figure 3b. The analysis of variance demonstrates significant differences in the averages for the three groups of spikes ( p < 10–4). A pairwise comparison of averages using the Mann–Whitney paired test also shows significant differences between all three groups of spikes on the basis of “tip area” ( p < 0.001). Thus, the splitting of the ears in F2 hybrids by the area of the awns is due to the fact that the tetraaristatum spikes have a larger area of the awns on average (Fig. 3b).

As for other characters of the spike, which demonstrate a monogenic 3:1 inheritance not for all projections, it is higher for the character associated with the area of the spike contour in the image (SA), which gives a split for three projections out of four. The perimeter of the spike contour (SP), its length (SL), the area of the quadrilateral model (q_S), and the length (q_L) demonstrate a close split to the 3:1 proportion for two projections of the spike out of four. It is possible that these characters are also controlled by a single gene, but the inaccuracy (ambiguity) of defining the parameters of the spike characters in the image may result in incomplete correspondence for different projections, making analysis more difficult.

## Discussion

The most important result of this investigation is the answer to the question of how much the results obtained by dif- ferent methods, namely, hybridological analysis and machine learning, coincide, and whether it is possible to judge the stage at which machine learning methods can be used in the context of working with species-specific characters.

Analysis of tetraaristatum. Among the studied hybrid plants, we obtain a split of 125 (normal) to 54 (tetraaristatum), χ2 3:1 = 2.549, p <0.05. The results are consistent with the hypothesis of monogenic inheritance of the tetraaristatum in a recessive manner. Earlier, in the works of E.F. Migushova and P.M. Zhukovsky (1969), M.A. Haque et al. (2011), R.V. Rozhkov (2014), and O.B. Dobrovolskaya et al. (2020), the recessive nature of the inheritance of this trait has been shown. The gene controlling the tetraaristatum trait, ta, is located in the long arm of chromosome 5A (Haque et al., 2011). Despite the fact that V.F. Dorofeev et al. (1979) noted the presence of tetraploid spike forms, in addition to T. carthlicum, in a number of subvarieties of the tetraploid species T. aethiopicum, most authors agree that the trait “tetraaristatum” is species-specific only for one tetraploid wheat species, namely for T. carthlicum (Haque et al., 2011; Goncharov, 2012; Dobrovolskaya et al., 2020).

In addition to tetraploid species, a trait phenotypically similar to tetraaristatum is characteristic of the hexaploid wheat species T. aestivum ssp. petropavlovskyi (Udacz. et Migusch.), however, in this case, it is a slightly elongated awn-like appendages on the spike glume (Goncharov, 2009). Nevertheless, we can argue that “tetraaristatum” is a species-specific trait only for T. carthlicum and can be effectively used for taxonomic purposes.

Color analysis of the spike. Among the studied hybrid plants, we obtain a cleavage of 144 (black + red spike) to 28 (white spike), χ2 13:3 = 0.689, p < 0.05. These results illustrate the phenomenon of dominant epistasis. The genes controlling spike coloration, Bg and Rg1, are located in chromosome 1AS, while Rg2 is located in chromosome 2BS. The digenic control of the red spike in wheat has been previously shown by a number of authors (Sobko, Sozinov, 1993; Kudryavtsev, Popova, 1994; among others).

Analysis of the type (character) of the awnedness. In the studied combination of F2 hybrids, there was a split of 46 plants with “T. aethiopicum-type” awns to 133 plants with “T. carthlicum-type” awns, which is consistent with the hypothesis of monogenic inheritance by the recessive type (χ2 = 0.047). The “awnedness type” trait is inherited monogenic, and the gene controlling it is located on chromosome 3A (Goncharov et al., 2003). The variation observed in this investigation in the severity of the “awnedness” trait (Fig. 3) is due to the presence of a significant number of modifier genes that control its severity (Sourdille et al., 2002; Wang et al., 2019). It is known that modifiers lead to “partial awnedness” (semi-awnedness, etc.) or changes in the length of the awns (from short to very long, exceeding the length of the spike) in some wheat varieties.

Analysis of the shape of the teeth on the spike glime. The presence/absence of a teeth on the spike glume is characteristic of all tetraploid wheat species, and the trait is not species-specific. However, it may have different degrees of expression in different varieties. According to the data obtained, the 175 (teeth present) to 10 (teeth absent) ratio corresponds to the 15:1 dihybrid inheritance hypothesis (χ2 = 0.225). The “Wheat manual book” (1980) states that both T. aethiopicum and T. carthlicum have a teeth, and that the shape of the teeth varies from short to long, and from blunt to sharp. However, only T. aethiopicum has a teeth that can be so long that it becomes an awn-like appendages.

Analysis of the hairy glume and the baldness in the trichomes on the spike glume. The hairy glume is an important taxonomic trait (Goncharov et al., 2007). It is controlled in tetraploid wheat species by the Hg gene, which is located in chromosome 1AS (Goncharov, 2012). In addition, for T. carthlicum, the absence of continuous hairy glume is shown, namely, the baldness in the trichomes on the spike glume in its lower part (Gandilian, 1972). Due to the different types of hairy glume, for the species T. carthlicum, trichomes with a baldness is a species-specific trait, and for their distinction, we have assigned the symbol “C” to the Hg gene according to the species symbols (HgC ). Considering these traits, we did not obtain reliable results for the 3:1 inheritance hypothesis, so additional research is required, as the severity is difficult to assess both visually and using AI.

Nevertheless, digital methods allowed us to evaluate the diversity of ears by 19 traits. And a much greater diversity of them in F2 hybrid plants was demonstrated than in the parental and F1 ones. It may be related to the presence of modifier genes (Bersimbaev, Shulembaeva, 2014). With the species-specific traits studied by us (Table 1), tetraaristatum, only one of the quantitative characters can be directly associated – the area of the awns in the image (SAA). The remaining 18 traits characterize the shape of the spike and its size and are not related to the color and glume traits. Our analysis of the mixture of Gaussian distributions showed that the F2 hybrid plants exhibit a 3:1 split into two classes based on the area of the awns, which is consistent with the tetraaristatum trait. Similar effects of population splitting into classes based on grain mass distribution have been described in triticale ×Triticosecale Wittm. ex A. Camus. (= syn. ×Triticale Tscherm.-Seys. ex Müntzing) (Kim et al., 2024). It should be noted that triticale is the result of intergeneric hybridization between wheat Triticum spp. and rye Secale cereale L. The splitting of different triticale varieties by grain weight was observed on different days of maturity (2–5 days after earing) for different seeding densities (150–300 kg/ha). However, the authors did not check the ratio of the number of plants in the grain weight classes. Our investigation demonstrates that a combination of automatic phenotyping methods and a mixture types of Gaussian distributions can, in principle, allow for automatic analysis of the separation of classes in F2 hybrids that differ in the values of phenotypes and exhibit a split depending on the inheritance model. This, in turn, can help identify genes associated with species-specific wheat plant traits.

## Conclusion

Based on the produced research, we can state that “tetraaristatum” is a species-specific traits only for T. carthlicum and can be effectively used for taxonomic, as well as in experiments using machine learning. The same applies to the nature of awnedness for T. aethiopicum.

The search for algorithms to solve the problem of automated classification of phenotypes in hybrid populations into classes continues (Merezhko, 2005; Rechkin, 2024). The method proposed in this investigation of using two Gaussian (normal) characters is effective for class distribution in oligogenic (mono- and di-genic) inheritance of traits.

## Conflict of interest

The authors declare no conflict of interest.
